# A new approach for efficient genotype imputation using information from relatives

**DOI:** 10.1186/1471-2164-15-478

**Published:** 2014-06-17

**Authors:** Mehdi Sargolzaei, Jacques P Chesnais, Flavio S Schenkel

**Affiliations:** Centre for Genetic Improvement of Livestock, Animal and Poultry Science Department, University of Guelph, 50 Stone Road East, Guelph, ON Canada; Semex Alliance, 130 Stone Road West, Guelph, ON Canada

**Keywords:** Family, Imputation, Haplotype, Rare variant, Sliding window

## Abstract

**Background:**

Genotype imputation can help reduce genotyping costs particularly for implementation of genomic selection. In applications entailing large populations, recovering the genotypes of untyped loci using information from reference individuals that were genotyped with a higher density panel is computationally challenging. Popular imputation methods are based upon the Hidden Markov model and have computational constraints due to an intensive sampling process. A fast, deterministic approach, which makes use of both family and population information, is presented here. All individuals are related and, therefore, share haplotypes which may differ in length and frequency based on their relationships. The method starts with family imputation if pedigree information is available, and then exploits close relationships by searching for long haplotype matches in the reference group using overlapping sliding windows. The search continues as the window size is shrunk in each chromosome sweep in order to capture more distant relationships.

**Results:**

The proposed method gave higher or similar imputation accuracy than Beagle and Impute2 in cattle data sets when all available information was used. When close relatives of target individuals were present in the reference group, the method resulted in higher accuracy compared to the other two methods even when the pedigree was not used. Rare variants were also imputed with higher accuracy. Finally, computing requirements were considerably lower than those of Beagle and Impute2. The presented method took 28 minutes to impute from 6 k to 50 k genotypes for 2,000 individuals with a reference size of 64,429 individuals.

**Conclusions:**

The proposed method efficiently makes use of information from close and distant relatives for accurate genotype imputation. In addition to its high imputation accuracy, the method is fast, owing to its deterministic nature and, therefore, it can easily be used in large data sets where the use of other methods is impractical.

## Background

The number of genotyped individuals is growing rapidly in both human and livestock populations due to the availability of affordable high density genotyping services. As a result, genomic information has grown in importance. Among genomic applications in livestock, especially in dairy cattle, genomic selection [1, 2] can substantially increase response to selection per unit of time compared to traditional selection [3]. Genomic selection has already been successfully adopted in the dairy cattle industry and has potential benefits for other livestock species e.g. [4]. However, genomic selection requires the routine genotyping of large number of young selection candidates, which can be expensive. To reduce genotyping costs, one option is to genotype young candidates with a cheaper lower density panel (LDP), which covers the genome uniformly, and to impute the genotype of untyped loci using information from a reference population genotyped with a higher density panel (HDP) [5, 6]. Sharing of genomic data in both human and livestock populations is of great advantage in order to increase reliability of predictions [7]. Imputation is also a powerful tool when combining data sets genotyped with different panels, provided enough overlap exists between panels. Sporadically missing genotypes can also be imputed in order to improve the genotype call rate [8].

Phasing and imputation methods can be broadly divided into family-based methods, which use linkage information from close relatives, and population-based methods, which use population linkage disequilibrium information [9]. Methods that rely on family information are mainly rule-based methods e.g., [10, 11]. They are reasonably accurate, especially if the LDP is sparse. Methods that use population information are usually probabilistic or model-based and exploit linkage disequilibrium between close SNP by modeling haplotype frequencies. Their accuracy depends mainly on panel density and reference size [12, 13]. Population imputation methods assume that individuals are unrelated. They do not make use of close relationships directly. However, they can still capture close relationships between individuals by finding long shared haplotypes [14]. Population-based methods are highly accurate, if both number of markers and number of reference individuals are high enough, but they are computationally intensive.

Kong et al. [11] presented a method to phase and impute long haplotype blocks. They used a group of surrogate parents (individuals of any sex that share IBD regions with the individual of interest) instead of true parents. In long range phasing, surrogate parents play a very important role when the true parents are not known/genotyped. This method was extended to use true parents when available [15]. Meuwissen and Goddard [16] proposed a combined family and population phasing and imputation method. First, family information is taken into account by an iterative peeling algorithm. In the second step, population information is used by approximating identical by descent probabilities.

Genealogy plays a very important role in phasing and imputation [11]. Real data usually shows a wide range of relationships between individuals from parent-progeny to individuals that are separated by many generations. At the haplotype level, close relatives share longer haplotypes that have lower frequency in the population. Distant relatives share shorter haplotypes which usually have higher frequency. Imputation and phasing are more accurate when using information from close relatives (i.e. long haplotypes with usually low frequency) than when using information from distant relatives. Therefore, one effective phasing or imputation strategy is to exploit the genealogy or relationships between individuals by searching for haplotypes from the longest to the shortest. This idea is a key aspect of the proposed method.

Accurate imputation of rare alleles is a challenging task. Rare alleles could contribute substantially to what is commonly called “missing heritability”, i.e. they could account for a substantial part of the genetic variance [17], although this is currently being debated. In addition, as minor allele frequency (MAF) decreases, association methods become more sensitive to genotyping errors. Therefore, accurate imputation of variants with low MAF is of importance and interest. Most rare variants (e.g. MAF <0.05) tend to be recent and are associated with longer haplotypes [18]. Therefore the use of information from close relatives is helpful for the imputation of rare variants.

In this paper a novel rule-based method for imputation is presented. The method relies on exploiting relationships between individuals assuming that close relatives share longer haplotypes while distant relatives share shorter haplotypes. The method has been successfully programmed in FImpute software. The performance of this method in terms of overall accuracy, accuracy of rare variants and computing requirements was investigated and compared to that of Beagle and Impute2.

## Results

The proposed method firstly uses the available pedigree information for accurate phasing and imputation, using an iterative approach. After family imputation, the remaining missing genotypes are imputed by an overlapping sliding window (OSW) approach, assuming that all individuals are related to some degree. With OSW approach, first more accurate information from close relatives is captured by moving long windows over a chromosome. Information from more distant relatives is then taken into account by making the window size shorter and shorter in each chromosomal sweep. For each window a haplotype library is built which is used for phasing and imputation within the window. The proposed method was compared to Beagle and Impute2 software on a large dairy cattle data set. The effect of genotyping close relatives (parents and grandparents) with HDP, different densities, and utilization of pedigree information was investigated (Table 1).

**Table 1:** **Scenarios used for the reference group to assess imputation accuracy**

Scenario	Structure of reference group	Reference size	Imputation method
**3 k/6 k to 50 k**			
A	Reference individuals were randomly selected after excluding parents and grandparents of the target group	100, 500, 1,000, 1,500, 2,000, 3,000, 5,000, 10,000	Population
B	All parents and grandparents of the target group	1,629	Population
C	As in B	1,629	Family + population
D	All males including sires and grandsires of the target group	64,429	Population
E	As in D	64,429	Family + population
**50 k to 300 k**			
F	As in A	100, 500, 1,000, 1,588	Population
G	Reference group consisted of all individuals	1,733	Population
H	As in G	1,733	Family + population

Because of its deterministic nature, the new imputation method was expected to be computationally faster than the Hidden Markov methods (HMM) used by software such as Beagle and Impute2. However, the first challenge for any imputation method is accuracy. Therefore, results of accuracy of imputation, as well as some other important aspects, such as the accuracy of imputation of rare variants, will be first presented followed by the computational efficiency.

### Overall imputation accuracy

**Figure 1:**
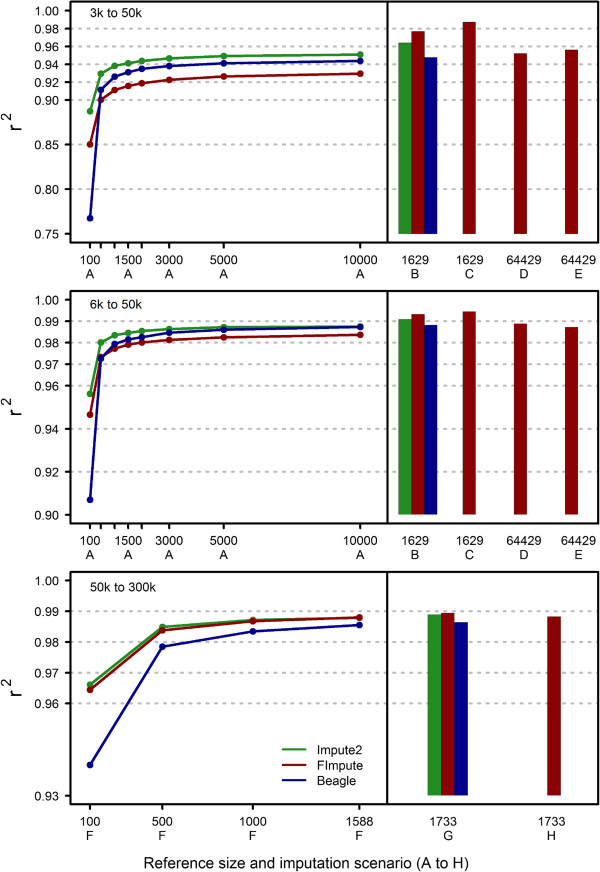
**Overall allelic r2 for FImpute, Beagle and Impute2 across different imputation scenarios.** There were 2000 and 500 young target individuals for imputation from 3 k/6 k to 50 k and from 50 k to 300 k, respectively. In scenarios A and F, reference groups with different sizes were randomly chosen after excluding parents and grandparents. The reference group in scenarios B and C included only parents and grandparents, in scenarios D and E it included all genotyped males and in scenarios G and H it included all genotyped individuals. Pedigree information was considered in scenarios C, E and H and was disregarded in scenarios B, C and G.

Allelic r2 for different scenarios and methods are presented in Figure 1. Allelic r2 is a measure of imputation accuracy that depends less on SNP allele frequency than concordance rate and it is calculated as the squared correlation between imputed and true genotypes [12]. In general, as expected, imputation was more accurate when the LDP was denser, the reference group was larger or when close relatives were included in the reference group.

In scenarios A and F, the goal was to assess the performance of FImpute with different reference sizes, and when target individuals were not closely related to those in the reference group. For these scenarios, parents and grandparents were excluded to reduce the chance of observing very long haplotype sharing between target and reference groups. FImpute achieved very high imputation accuracy for 6 k to 50 k and 50 k to 300 k. Allelic r2 for 3 k to 50 k was relatively high, but not as high as those achieved by Beagle and Impute2. FImpute performed much better for denser panels, because it could find shared haplotypes between distant relatives with greater precision. For 50 k to 300 k, the allelic r2 attained with FImpute and Impute2 were similar across different reference sizes and were higher than those attained with Beagle. Allelic r2 was higher with a larger reference size and the gain in accuracy for FImpute and, especially, Beagle was larger than for Impute2. This might be due to the fact that Impute2 samples a fixed number of haplotypes (default settings are 80 for reference and 500 for target) from approximated surrogate family members, regardless of the number of haplotypes in the reference group [19]. As can be seen from Figure 1, one can expect that Beagle will be similar in performance to Impute2 if the reference size is big enough.

For 3 k/6 k to 50 k cases, all target individuals had genotyped parents and grandparents, therefore scenarios B without pedigree information and C with pedigree information were designed to investigate the impact of close reference relatives on allelic r2 and efficiency of FImpute for this situation. Only parents and grandparents were included in the reference group. Inclusion of very close relative in the reference group substantially increased the imputation accuracy especially when pedigree information was taken into account (Scenario C). Browning and Browning [14] also found that when parents were included in the reference group, phasing accuracy using population haplotype frequency information was substantially higher. The increase in imputation accuracy was more evident for 3 k to 50 k. However, as the panel becomes denser the importance of having reference individuals with close relationships to the target animals decreased. In scenario B, FImpute always gave higher allelic r2 compared to Beagle and Impute2. This indicates that FImpute can better capture information from close relatives even without a known pedigree. Impute2 performed better than Beagle in scenario B.

For imputation from 50 k to 300 k, most parents and grandparents of the target group were not genotyped with the 777 k panel, therefore, it was not possible to try scenarios similar to B or C. Instead, all animals including genotyped parents and grandparents of those in the target group were included in the reference group, and imputation was carried out with and without pedigree information (scenarios G and H). The gain in accuracy due to adding close relatives in the reference group was small (scenario G). The use of pedigree information had a slightly detrimental effect on the allelic r2 (scenario H). This slight decrease in accuracy when pedigree information was used could be due to errors in the pedigree. Such errors cannot be identified for ungenotyped individuals. This result, however, indicates that the OSW approach is more robust than family imputation for high density imputation. With a reference group that includes genotyped parents and grandparents, FImpute showed slightly more gain in allelic r2 than Beagle and Impute2.

In cattle, males tend to contribute more genetically to the population than females. Semen samples are usually available for older males and can be used for genotyping while the genetic material of older females is most often not available. Scenarios D and E were designed to investigate imputation based on a male-only reference group. The reference for imputation from 3 k/6 k to 50 k consisted of all 50 k genotyped males including sires and grand sires. The size of this reference group was 64,429 males. Imputation with Beagle and Impute2 was not feasible for this scenario due to their high computational demand. The allelic r2 for FImpute in scenario D (no pedigree information) was 0.952 for 3 k to 50 k and 0.989 for 6 k to 50 k. These values were higher than those of scenario A with 10,000 reference individuals. The higher accuracies for scenarios D and E were mainly due to the larger reference population size and the presence of sires and grand sires in the reference group. One conclusion from comparing scenarios B and C to scenarios D and E is that for low density imputation (especially 3 k or sparser to 50 k), the genotypes of female ancestors and the availability of pedigree information are very important in order to achieve optimal imputation accuracy.

### Imputation accuracy of rare variants

**Figure 2:**
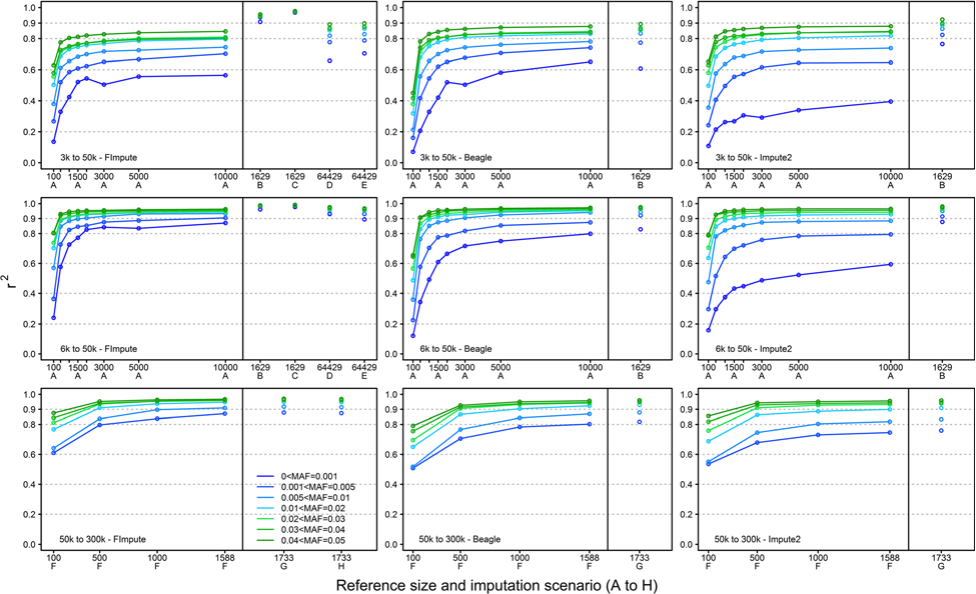
**Rare allele imputation: allelic r2 in different MAF bins for FImpute, Beagle and Impute2.** There were 2000 and 500 young target individuals for imputation from 3 k/6 k to 50 k and from 50 k to 300 k, respectively. In scenarios A and F, reference groups with different sizes were randomly chosen after excluding parents and grandparents. The reference group in scenarios B and C included only parents and grandparents, in scenarios D and E it included all genotyped males and in scenarios G and H it included all genotyped individuals. Pedigree information was considered in scenarios C, E and H and was discarded in scenarios B, C and G.

Accurate imputation of SNP with rare alleles (MAF ≤ 0.05) is important since rare alleles may account for a large portion of the genetic variation that is not explained by common alleles [20]. The relationship between allelic r2 and MAF in the target group is illustrated in Figure 2 for different scenarios. In general, allelic r2 increased as MAF increased for all methods. The gain in the imputation accuracy of rare variants increased with reference population size and panel density. From Figure 2 the imputation of rare alleles is more sensitive to the size of the reference group compared to the imputation of common alleles. The larger the reference group size, the more accurate the imputed genotypes for SNP with low MAF (≤0.05). For scenarios A and F, where close relatives were excluded from the reference group, FImpute was able to call SNP with low MAF with higher accuracy. Because most rare variants are recent and located on long haplotypes, this shows that FImpute can exploit longer haplotypes (of closer relatives) quite efficiently. Accuracy of imputation for SNPs with low MAF was consistently higher for FImpute than for Impute2 in all scenarios. Accuracy was also higher than for Beagle for 6 k to 50 k and for 50 k to 300 k. For the imputation of rare variants, Impute2 was always inferior to FImpute and to Beagle despite the fact that Impute2 gave very high overall accuracy for scenarios A and F. Impute2 would need a much larger reference group to achieve the same level of accuracy as FImpute or Beagle. One could potentially try to increase the number of sampled haplotypes for Impute2, but this would require increased computing time.

In scenario B, when only parents and grandparents were allowed in the reference group with no pedigree information, the accuracy of FImpute for rare variants increased considerably. Similar to overall allelic r2, the most gain in accuracy was observed for a sparse panel (3 k to 50 k), showing the importance of close relatives or longer haplotypes for imputation of sparse panels. Under scenario B, allelic r2 of rare variants from Impute2 and Beagle also increased but they did not reach the level obtained by FImpute. However, Impute2 exploited the close relationships better than Beagle did. When, in addition to the use of parents and grandparents in the reference population, pedigree information was used for imputation (scenario C), FImpute reached very high accuracy (0.968 – 0.992) for rare alleles. When the reference group consisted of all males, including sires and grandsires (scenario D), rare variants were imputed with high accuracy from 6 k to 50 k. The accuracy of rare variant imputation from 3 k to 50 k was moderate, but still higher than in scenario A with 10,000 reference individuals.

For imputation from 50 k to 300 k, adding genotyped parents and grandparents into the reference group with or without pedigree information (scenarios G and H) did not result in a substantial gain in accuracy of imputation of rare alleles. This is because with a high density panel, shared haplotypes between distant relatives can be found more easily and accurately. Therefore, for imputation of high density panels, as long as the reference group is large enough and moderately related to the target group, immediate relatives play much less of a role in imputation.

### Computational performance

**Figure 3:**
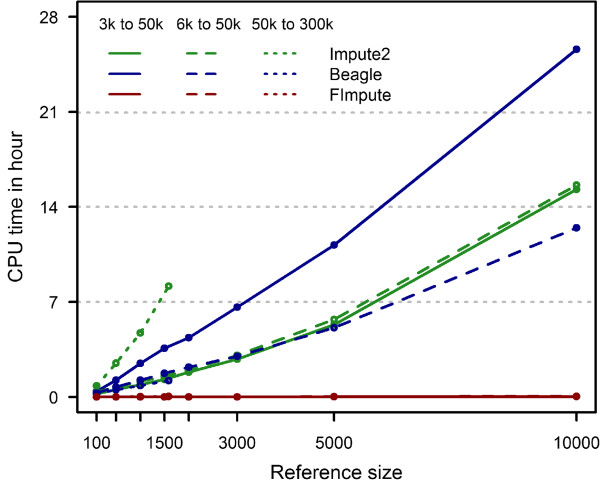
**CPU time for Beagle, Impute2 and FImpute over different reference sizes.** No pedigree information was used and genotyped parents and grandparents were excluded.

Figure 3 illustrates the CPU time for imputation of chromosome 15 for each of the three methods. Chromosome 15 has an average length for an autosome (84 Mb). FImpute was considerably faster than the two other methods for all scenarios. For example, for 2,000 target and 10,000 reference individuals and for imputation from 6 k to 50 k, FImpute took 3 minutes to completion while Beagle took more than 15 hours and Impute2 more than 12 hours. Computing time of all three methods increased as the reference size increased. For FImpute and Beagle, computing time increased linearly with increasing reference size, , but the increase was more rapid for Impute2. However, while FImpute and Impute2 became slower with denser panels, Beagle became faster as the density of the LDP increased.

With rapid advances in CPU technologies, multi-core CPUs are becoming standard. Parallel processing on a multi-core system can make the imputation process substantially faster. Existing imputation software (e.g. Beagle and Impute2) could be modified to take full advantage of multi-core processing. The current version of FImpute is able to parallelize chromosomes on multi-core systems.

## Discussion

A new method for genotype imputation was presented in this paper. The method is deterministic, and essentially searches for long to short haplotypes, which represent close to far relationships, respectively. Pedigree information is taken into account if known. The method makes no specific assumption about the degree of relationship between individuals. Similar to the long-range phasing algorithm [11], the new method initially identifies long shared chromosomal segments. However, the method is fundamentally different from that of Kong et al. [11], because it is not iterative, does not create surrogate family members, works with haplotypes instead of genotypes, and searches for short shared haplotypes as well as long ones. One key task in the new method is finding the beginning and the end of shared haplotypes between individuals. To do so, a chromosome is swept with sliding windows of different sizes starting with a long window and gradually shrinking it. Sliding windows are overlapped in order to facilitate the search for the beginning and end of shared haplotypes. The imputation accuracy of the new method was very high despite the fact that no posterior distribution is sampled. This was primarily due to the availability of high density panels with high genotype quality, which together allow for accurate haplotype matching. The current genotyping technologies such as Illumina Infinium are very accurate, with a reproducibility greater than 99.9% [21].

The existence of family information, especially knowledge of the sire and dam, is very important for low density phasing, shown here for imputation from 3 k to 50 k. As expected, imputation from denser SNP panels leads to higher accuracy because the OSW approach can find shorter haplotypes from distant relatives with higher precision. As a result, it is less dependent on the availability of close family information. For imputation from 50 k to 300 k, there is a slight decrease in allelic r2 when the pedigree information is used (scenario H) compared to a situation with no pedigree information (scenario G). This decrease can be attributed to pedigree errors for ungenotyped animals. When the pedigree is not traced for ungenotyped animals, the level of imputation accuracy is the same for scenarios G and H. With a dense marker panel, family phasing and imputation do not provide much gain over the OSW approach alone. For imputation from 50 k to 300 k, having close relatives in the reference group could lead to a higher gain in imputation accuracy than that observed in scenario G, when the reference and target groups are distantly related, as might occur in the study of some human populations. In all cases, family information remains important for the correction of genotyping errors.

A software program, FImpute, was developed based on this new method, and the results compared to two well established imputation methods in human genetics, Beagle and Impute2. FImpute was not compared to other methods since other studies have already shown the superiority of FImpute, Beagle and Impute2 [22, 23]. Beagle and Impute2 assume that individuals are unrelated. They model haplotype frequency and use the hidden Markov model to calculate a posterior distribution. The relationship between reference and target groups significantly influences phasing and imputation performance [14]. Results in this paper show that the OSW approach is able to exploit close relationships more efficiently than Beagle and Impute2 in all scenarios, especially when the LDP was the sparsest (i.e. imputation from 3 k to 50 k). This is because FImpute starts with highly accurate haplotype matches, corresponding to the long haplotypes of close relatives. The first window covers the whole genome so only parent-progeny matches are found. Impute2 was superior to Beagle in this regard, likely due to the selection of surrogate family members, which carry long shared haplotypes, for haplotype sampling [19].

For imputation from 3 k to 50 k, imputation accuracy was moderate. However, the size of the reference group was more important. In contrast to population model-based methods, FImpute can handle a very large reference size. Therefore, an additional scenario with 108,755 reference individuals (all the available 50 k individuals, excluding parents and grandparents of the target group) and no pedigree information was considered. The allelic r2 from FImpute was 0.943 for 29 autosomes and the required computation time was 53 minutes for chromosome 15. Handling such large reference groups is not possible within a reasonable time limit for Beagle and Impute2, therefore a comparison was not attempted.

One of the most challenging tasks is the imputation of rare variants. Accurate imputation of SNP with rare alleles (MAF ≤0.05) is important especially when the imputed genotypes are to be used in association studies. FImpute imputes rare alleles with high accuracy because it is efficient at finding the long haplotype matches on which rare alleles are most likely located [18]. Impute2 and Beagle impute rare variants with lower accuracy, except for population imputation from 3 k to 50 k with Beagle. In an independent study, FImpute had higher allelic r2 for rare variants than Beagle and Impute2 [23]. In our study, except for very small reference groups, Beagle performed better than did Impute2 for rare variants. This finding was in contrast with that of Howie et al. [13].The difference might be due to: 1) improvements in Beagle's methodology since 2009, 2) different population structures, 3) different SNP density and 4) the fact that Impute2 restricts phasing and imputation updates to 500 template haplotypes (default setting), which could reduce sampling space if haplotype diversity is high. On the other hand, Impute2 tends to impute common variants with slightly better accuracy than Beagle and FImpute.Comparing scenarios A, B, C, D and E against each other (Figure 2) suggests that the genotypes of both parents are very helpful for obtaining high imputation accuracy, especially for rare variants, and that the direct use of pedigree information is beneficial. The gain in accuracy of rare variants was more pronounced with sparser panels. In livestock populations, only elite dams are genotyped with a HDP, and most other dams and young females are genotyped with a LDP, for economic reasons. To investigate the benefit of obtaining low density genotypes on dams versus not genotyping them, two additional scenarios similar to scenario C were considered, where all the dams and grand-dams were either ungenotyped or genotyped with the 3 k panel, and where the reference group included only 251 sires and grandsires. Despite the small reference group size, overall allelic r2 for these two scenarios were 0.934 and 0.953, showing the importance of genotyping dams with a LDP to increase accuracy in this situation. However, the accuracy of imputation of SNP with rare alleles was low for both scenarios, mainly due to the small reference size. For example, for the SNP group with MAF between 0.001 and 0.005, allelic r2 was 0.550 with ungenotyped dams and 0.607 with 3 k dams. The gain in accuracy of SNP with rare alleles (<0.05) ranged from 0.037 to 0.063. Therefore, when the LDP is sparse, it is important to include dams with low density genotypes in the target group.

In dairy cattle selection schemes, selection intensity is high and usually only a few top sires are used to produce the next generation. This intense selection over the past decades has resulted in a lower effective population size and consequently in a high level of LD in dairy cattle breeds [24]. FImpute is well suited to such situations, because it assumes that individuals are related and exploits relatives’ information from the closest to the farthest. The presented method has not been tested on human population, where the effective population size is larger and reference individuals are usually genetically more distant from the target group. A separate study is needed to assess the performance of FImpute on human data.

Another notable feature of FImpute is low computational requirements. Current routine imputation in dairy cattle in North America includes close to 360,000 animals with 5 different LDPs and a very large reference size of close to 30,000 parents genotyped with 50 k. These numbers are expected to grow fairly rapidly over time. Beagle or Impute2 cannot handle this situation in a reasonable time frame, while FImpute can do it in less than 3 hours. An alternative combined family and population imputation method, which can quickly perform large-scale imputation, is findhap [25]. However, it was shown that, compared to FImpute, findhap yields lower imputation accuracy when close relatives are not genotyped with HDP [23]. Another computationally fast method for large-scale imputation is PedImpute [26]. However, the underlying methodology in PedImpute is similar to findhap [26], so they can be seen as one method with different implementations. Pre-phasing has been suggested to speed up the imputation process [27, 28]. To this end, haplotypes are constructed once and stored so they can be used for subsequent imputations. While this strategy might work for human genomic studies due to denser SNP panels and sparser relationships between individuals, it is not well suited to livestock applications where LDPs are sparse and the genotypes of parents of young animals are continually added to the reference group. In such a case, the use of pre-phased haplotypes will not lead to optimal imputation accuracy for the target group. Generally, pre-phasing can only be effectively implemented in situations where individuals newly genotyped with the HDP are not closely related to the target individuals. FImpute has the capability to use pre-constructed haplotypes. However, for livestock populations, the use of pre-phased haplotypes for imputation is only recommended when the LDP has a high density. Even then, in livestock species, reducing the reference population to a group of animals that have high genomic relationships with the target individuals might be a better strategy than using pre-constructed haplotypes, and is an approach that warrants further investigation.

## Conclusions

In this study an accurate and fast imputation method was presented. The method is based on the concept that close relatives share long haplotypes, while distant relatives share short haplotypes. Because there are more markers on longer haplotypes, accuracy of imputation from long haplotypes is higher compared to short haplotypes. Therefore, to achieve high accuracy, imputation is carried out using overlapping sliding windows starting with long haplotypes and moving towards short haoplotypes. The results indicated that the presented method is competitive with existing well-established imputation methods in terms of overall accuracy and yet it is computationally very efficient and can handle very large data sets, which are encountered in livestock species.

## Methods

### Family phasing and imputation

The length of the haplotypes shared by two individuals on a specific chromosome is a function of the number of crossovers that occurs in the genealogical path that connects them. This path might be known for close relatives that share long haplotypes, but unknown for distant relatives that share short haplotype segments.

Even when pedigree information is not available, family information can be captured by searching for long haplotypes [11]. However, the use of pedigree information can result in more accurate phasing, especially when LDP is sparse, due to better crossover resolution [24, 15, 29]. As the density of the panel increases, the importance of pedigree information decreases. This is because higher density increases the likelihood of finding correct shared haplotypes, especially for short segments, and increases crossover resolution. An efficient rule-based family phasing algorithm that takes into account paternal half-sib family information was presented previously in Sargolzaei et al. [24]. The algorithm is iterative and, in each iteration, it accumulates the relative information by tracing the pedigree up and then down. This algorithm has been modified to accommodate maternal information and is described in the Appendix.

**Figure 4:**
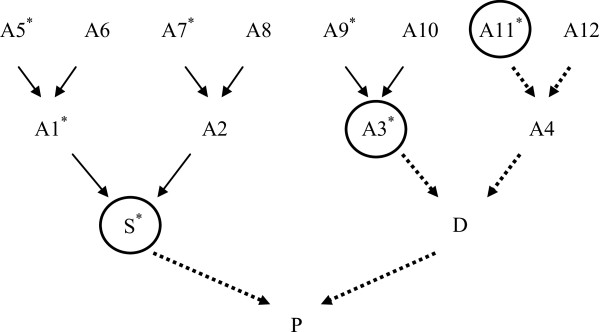
**Tracing genotyped individuals for family imputation.** A1, … A12 represent ancestors of individual P, and S and D are its sire and dam. An asterisk indicates that the individual is genotyped. The dotted line shows the traced path for animal P.

When both parents are genotyped with HDP and their haplotypes are reconstructed, the imputation is straightforward. The haplotypes of progeny are matched against parental haplotypes, and missing information is filled based on the detected match. When a crossover is detected it is assumed that the most likely position of the crossover is in the middle of the two consecutive SNPs. However, to prevent introducing errors, if the distance between the two SNPs is larger than 2 cM, then only 1 cM plus 30% of the extra distance is filled in from each side.When at least one parent is not genotyped, the pedigree of the ungenotyped parent is traced back to find genotyped ancestors. For the genotyped ancestors, the pedigree is not traced because the older genotyped ancestors do not provide additional information. Parents with unphased genotypes are considered not genotyped. For sex-specific chromosomes, the sires of male individuals are set to unknown. After a haplotype match is found, 5% is trimmed from each side of the haplotype to reduce the errors caused by random matches at the edges. Figure 4 shows how the tracing algorithm works when one parent is ungenotyped. In this example, genotyped ancestors are marked with an asterisk. The paternal haplotype of the progeny (P) is matched against the sire's (S) haplotypes and the maternal haplotype of P is matched against ancestors A3 and A11. If shared haplotypes overlap between A3 and A11, the longest haplotype is accepted for the overlapping segment.

For individuals with one missing parent, one haplotype is imputed and the other haplotype is processed with population imputation as described below.

### Population phasing and imputation

The relationship between two individuals due to common ancestors is a function of the average length of shared haplotypes based on marker information. The length of the haplotype segments depends on the number of crossovers that occurred since the common ancestor. Closer relatives usually share longer haplotypes while more distant relatives share shorter haplotypes. Haplotypes tend to become shorter over generations mainly due to recombination and mutation. The shared haplotypes arising from recent crossovers or mutations are long [18] and can be seen only between close relatives. Furthermore, the accuracy of a haplotype match between two individuals is mainly a function of the length of the shared haplotype and the number of matched SNP. The longer the shared haplotype, the more accurate the match [11]. However, when more than one match is found, the match with higher frequency is considered the most likely one. Therefore, searching for matches from long to short haplotypes using a sliding window approach should lead to accurate phasing and imputation, provided there are enough markers on the panel. One challenge is finding the beginning and end of the shared haplotype between individuals. If a chromosome is split into a fixed number of segments then some haplotype matches may be missed because they do not fit within the defined window. Another challenge is to keep consistency of haplotype phase across sliding windows. To overcome these issues one can allow the sliding windows to overlap in order to increase the chances of finding the correct haplotype matches. With the overlapping sliding window (OSW) approach, a chromosome or specific genomic region is swept many times starting from a long window size and slowly moving to a short window size. A fixed window size is applied within each sweep. The window size is shrunk by factor of 0.1 - 0.2 after each sweep. Optimum overlap between windows is set at 0.6 - 0.7, based on empirical results from real data. The maximum window size is set to 1,000 SNPs and the minimum to 2 SNPs.

If enough family information is available, individuals with high density genotypes and reconstructed phase from family information can be added to the reference group, which increases the overall accuracy of imputation. Following is the detailed algorithm of OSW for the situation where imputation cannot be done with family information:

- The first window covers the whole genome and this full window identifies progeny-parent pairs (i.e. parentage discovery)
- Process chromosome by chromosome
- Process from high density genotype group to low density genotype group
- Sweep the chromosome starting with the maximum window size (1,000 SNP)
- Build a haplotype library based on phased genotypes, including haplotypes reconstructed from family information. If phase of a genotype is ambiguous, treat it as missing
- Find similar haplotypes (≥99% similarity) in the current haplotype library based on already phased genotypes (including homozygotes), infer haplotypes for heterozygotes if possible, merge similar haplotypes, and calculate haplotype frequencies
- For individuals of the current density group and window size,
- if there are any unphased and missing genotypes and a pair of matches with similarity ≥ 0.99 in the haplotype library is found, phase the unphased genotypes and impute (one of the haplotype matches could be mosaic)
- If the window size exceeds the minimum size (2 SNP), shrink the window and go to the next window
- Impute the remaining missing genotypes by random sampling of alleles based on the frequencies calculated in the reference group

Since accuracy of phasing is higher for larger window sizes, these accurate phases act as anchors for haplotyping in smaller windows. Therefore, the switch rate between haplotypes from different windows tends to be minimized. More phasing errors at the beginning and end of segments can be expected, therefore, a portion corresponding to 5% from the beginning and end of each segment is not phased or imputed.

When the LDP is relatively sparse and there are close relationships between individuals (i.e. parent-progeny), the whole genome window is important. Therefore, with a sparse LDP, all chromosomes must be analyzed together in order to achieve optimal performance, while with a denser LDP the whole genome window may be skipped. Since individuals are unphased for the first window, only homozygous loci are considered in order to avoid unnecessary computation.

The method is rule-based and, therefore, computationally efficient for very large reference groups and high density panels. The proposed method has been implemented in a software package called FImpute and it is freely available for research purposes at http://www.aps.uoguelph.ca/~msargol/fimpute.

### Performance assessment

The imputation accuracy of the new method was assessed using a North American Holstein data set consisting of the data used for official genomic evaluations in Canada in April 2013. The data set was provided by the Canadian Dairy Network (CDN, Canada) and contained 2233, 112738, 3979, 50768 and 90241 animals genotyped with the 777 k, 50 k, 80 k, 8 k and 6 k chips from Illumina Infinium SNP array, respectively. There were also 49,334 animals genotyped with the 3 k chip from Illumina Golden Gate array. For this study, only animals genotyped with 50 k and 777 k panels were used, and imputation from 3 k to 50 k, 6 k to 50 k and 50 k to 777 k was investigated by simulating a LDP for target animals genotyped with a HDP. Animals and SNP with low genotype quality were already filtered by the Animal Improvement Program Laboratory (AIPL, USA) and CDN. Details of quality control measures are given in VanRaden et al. [30]. In this study, only the SNPs over the 29 autosomal cattle chromosomes were considered. The final number of SNPs and the overlap between panels are shown in Table 2. After edits, the number of SNPs on the 777 k panel was greatly reduced (mainly due to the removal of SNPs with a high level of LD with other SNPs). Therefore, this panel is referred to as the 300 k panel in this paper. There were 4,023 SNPs on the 50 k panel that were not on the 300 k panel, and therefore were excluded for imputation from 50 k to 300 k.

**Table 2:** **The number of SNP on each panel (diagonals) and number of overlapping SNP between panels (off diagonals)**

Panel	3 k	6 k	50 k	300 k
3 k	2,485	-	-	-
6 k	2,485	6,603	-	-
50 k	2,485	6,603	44,369	-
300 k	22,75	6,556	40,346	301,318

The pedigree information of progeny was removed if there was more than 2% of Mendelian inconsistencies between the genotypes of parents and progeny. All pedigree information, including ungenotyped animals, was taken into account for family imputation.

In most livestock applications, younger individuals are genotyped with a LDP for genomic selection. Therefore, for imputation from 3 k/6 k to 50 k, the 2,000 youngest 50 k animals with genotyped parents and grandparents were considered as the target group, i.e. the group of animals used to validate the accuracy of imputation. For imputation from 50 k to 300 k, only the 500 youngest animals were chosen as the target group. Among these, there were 7 animals with two genotyped parents, 247 with one genotyped parent and 246 with no genotyped parent. However, 438 animals had at least one genotyped grandparent. Genotyped parents and grandparents are helpful in assessing accuracy of family imputation. For target animals, the LDP (3 k, 6 k or 50 k) was simulated by masking the true genotypes, i.e. removing all SNPs present on the HDP but not present on the LDP. Different sizes and structures for the reference group were used as described in Table 1 in order to explore the performance of the proposed method in various situations. For scenarios A and E, parents and grandparents of the target animals were removed for better assessment of population imputation. In dairy cattle, most males are genotyped with a HDP and most females with a LDP. Therefore in scenario D all males were considered to be in the reference group.

The accuracy and computational performance of the proposed method (FImpute) were compared to Beagle version 3.3.2 and Impute2 version 2.3, which are commonly used imputation methods. There are other accurate population-based imputation software such as Phase, fastPhase [31] and MaCH [32], which were not included in this comparison due to their very high computational demands. Due to the high computational demands of Beagle and Impute2, the comparison was not practical for scenario D. Default settings were used for all the software, except for effective population size (Ne) in Impute2, which was set to 80, based on an estimate of effective population size obtained in Sargolzaei et al. [24]. The main default settings were niterations = 10 and nsamples = 4 for Beagle, -iter 30, -k 80 and -k_hap 500 for Impute2 and sw_shrink_factor = 0.15 and sw_overlap = 0.65 for FImpute. Programs ran on a Linux server with two E5-2690 Intel Xeon processors each with 8 cores and 16 logical processors clocked at 2.9 GHz and with 132 GB RAM memory.

To achieve high imputation accuracy, chromosomes were not split into smaller chunks. Each single run of Beagle and Impute2 imputes one chromosome, while all the chromosomes were considered in a single FImpute run.

Imputation of target groups was performed with and without pedigree information to assess the robustness of the proposed method with population imputation only, or with both family and population imputation. The later, however, is recommended for most applications. Allelic r2, the squared correlation between imputed genotypes and true genotypes [12], was used as a measure of imputation accuracy. Concordance rate was not used since this measure does not adequately reflect the imputation accuracy of SNP with a rare allele.

## Appendix

### Family phasing algorithm

The following algorithm makes use of information from parents and relatives to estimate a probability (*P*_*ij*_) that an animal *i* inherited allele 1 from its father at locus *j*.

Let genotype codes 0, 1, 2 and 5 denote A2A2, A1A2, A1A1 and missing, respectively, and let subscript i denote animal i and subscript j denote marker j.

Remove first any progeny-parent's Mendelian inconsistencies

**Initialization**. Process individuals from the oldest to the youngest:

Process SNPs

Set *P*_*ij*_ to 0, 0.5 or 1 when own genotype is 2, 1 or 0, respectively.

for 0 < *P*_*ij*_ < 1 (heterozygous):

if sire genotype is 2 or 1, set *P*_*ij*_ to 0 or 1, respectively.

else if dam genotype is 1 or 2, set *P*_*ij*_ to 0 or 1, respectively.

if sire and dam are unknown or ungenotyped set *P*_*ij*_ to 1 at the first heterozygous marker on each chromosome.

*K* = 0.15 is a threshold value for partial informativeness of a heterozygous marker

**Step 1.** Update parent phases using progeny information:

Process parents from the youngest to the oldest

Process heterozygous SNP only

if phase is uncertain (0 < *P*_*ij*_ < 1) then

find the nearest partially informative heterozygous SNP on both sides that is, |*P*_*ij* ′_ - 0.5| > *K*,

compute effective number of non-recombinant progeny, *z*, at *j* and *j*′ as:

if the parent is a sire, set *PP* to *P* and if the parent is a dam, set *PP* to 1 – *P*

Then update *P*_*ij*_ as follows:


, where *d* is distance in Morgan between markers *j* and *j*′


**Step 2.** Update progeny phases using parent information:

Process individuals from the youngest to the oldest (*i* = 1 to *n*)

Process sire and then dam

Process progeny's heterozygous SNP only (*j* = 1 to *nSNP*)

if phase is uncertain (0 < *P*_*ij*_ < 1) then

if the parent is a sire, set *PP* to *P* or if the parent is a dam, set *PP* to *P* for homozygous loci and to 1 - *P* for heterozygous loci

find the nearest partially informative SNP on the left (L) and on the right (R) for the progeny when the parent (*m*) is heterozygous. That is, for left marker, |*X*_*L*_ - 0.5| > *K*, where *X*_*L*_ = *PP*_*iL*_*P*_*mL*_ + (1 + *PP*_*iL*_)(1 - *P*_*mL*_) The Same is used for R SNP.

if left and right SNPs are found then compute


else if one marker on the left found then compute


else if one marker on the right found then compute


Update *P*_*ij*_ as follows:

if the parent is a sire, set *PP* to *P* or if the parent is a dam, set *PP* to *P* for homozygous loci and to 1 - *P* for heterozygous loci


Repeat steps 1 and 2 until the sum of squared changes in *P*_*ij*_ is sufficiently small. Based on a simulation study *P* values were stabilized after 8 to 10 iterations. Finally, for heterozygous loci *P*_*ij*_ < 0.5 indicates that allele 1 is from the father and allele 2 is from the mother and the other way around for *P*_*ij*_ > 0.5. In order to save memory, haplotypes are coded as 3 when *P*_*ij*_ < 0.5 and as 4 when *P*_*ij*_ > 0.5.
